# Body mass index and clinical response to intravenous or subcutaneous abatacept in patients with rheumatoid arthritis

**DOI:** 10.1007/s10067-017-3788-1

**Published:** 2017-08-18

**Authors:** Maria-Antonietta D’Agostino, Rieke Alten, Eduardo Mysler, Manuela Le Bars, June Ye, Bindu Murthy, Julia Heitzmann, Radu Vadanici, Gianfranco Ferraccioli

**Affiliations:** 1 Departement de Rhumatologie, AP-HP, Hôpital Ambroise Paré, INSERM U1173, Laboratoire d’Excellence INFLAMEX, UFR Simone Veil, Versailles-Saint-Quentin University, 92100 Boulogne-Billancourt, France; 2 Schlosspark-Klinik University Medicine, Berlin, Germany; 3 Organización Médica de Investigación, Buenos Aires, Argentina; 4 Bristol-Myers Squibb, Rueil-Malmaison, France; 5 Bristol-Myers Squibb, Princeton, NJ USA; 6 Excelya, Boulogne-Billancourt, France; 7 Catholic University of the Sacred Heart, Rome, Italy

**Keywords:** Body mass index, DAS28, Disease activity, Pharmacokinetics, Rheumatoid arthritis

## Abstract

This post hoc analysis of ACQUIRE (NCT00559585) explored the effect of baseline body mass index (BMI) on the pharmacokinetics of and clinical response to subcutaneous (SC) or intravenous (IV) abatacept in patients with rheumatoid arthritis (RA). ACQUIRE was a phase 3b, 6-month, double-blind, double-dummy study in which patients with RA were randomized (1:1) to SC (fixed - dose; 125 mg/week) or IV (weight-tiered; ~ 10 mg/kg/month) abatacept plus methotrexate. In this analysis, minimum abatacept plasma concentration (C_min_) was measured at 3 and 6 months, and clinical remission over 6 months was assessed by Disease Activity Score 28 (C-reactive protein; DAS28 [CRP], < 2.6), Simplified Disease Activity Index (SDAI, ≤ 3.3), and Clinical Disease Activity Index (CDAI, ≤ 2.8). Data were stratified by baseline BMI (underweight/normal, < 25 kg/m^2^; overweight, 25 to < 30 kg/m^2^; obese, ≥ 30 kg/m^2^) and administration route. Of the 1456/1457 patients for whom baseline BMIs were available, 526 (36%; SC 265, IV 261) patients were underweight/normal, 497 (34%; SC 249, IV 248) were overweight, and 433 (30%; SC 221, IV 212) were obese. Median C_min_ abatacept concentration was ≥ 10 μg/mL (efficacy threshold) at 3 and 6 months in > 90% of patients across BMI groups with both administration routes. DAS28 (CRP), SDAI, and CDAI remission rates at 6 months were similar across BMI groups and 95% confidence intervals overlapped at all time points in both separate and pooled SC/IV analyses. Therapeutic concentrations of abatacept and clinical remission rates using stringent criteria were similar across patient BMIs and administration routes.

## Introduction

More than 60% of patients with rheumatoid arthritis (RA) are classified as overweight or obese according to body mass index (BMI; > 25 kg/m^2^) [1, 2]. Adipose tissue is known to have pro-inflammatory properties [3]; however, its role in the development and severity of RA is unclear. Whereas women who are overweight or obese have been found to be at increased risk of RA at a younger age [4], a higher BMI has been independently associated with a lower risk of structural damage progression [5]. A meta-analysis found that Disease Activity Score in 28 joints (DAS28) and functional disability (Heath Assessment Questionnaire) score were both significantly higher in patients with RA who were obese (BMI > 30 kg/m^2^) versus non-obese (BMI ≤ 30 kg/m^2^); conversely, radiographic progression was negatively associated with obesity (*p* < 0.05) [6].

BMI is a known predictor of treatment response and is likely to be a consideration in the development of an optimally effective, personalized treatment plan for a patient with RA. An epidemiological study showed that, following 6 months of treatment with conventional synthetic disease-modifying antirheumatic drugs (csDMARDs), patients with a BMI ≥ 25 kg/m^2^ had a > 50% lower chance of achieving a good response and a > 40% lower chance of achieving remission than patients with a BMI < 25 kg/m^2^ [7]. Similarly, a recent meta-analysis of 3368 adults with RA showed that, following treatment with either biologic DMARDs (bDMARDs) or csDMARDs, patients who were obese (BMI ≥ 30 kg/m^2^) were 40% less likely to have achieved disease remission and 50% less likely to have achieved sustained remission than those who were non-obese (BMI < 30 kg/m^2^) [8].

The extent to which response to treatment is influenced by patient BMI varies between bDMARDs and may be linked to their mode of action. For the tumor necrosis factor inhibitors (TNFis), there is considerable evidence for lower efficacy in obese than in non-obese patients with RA [9]. In one study, remission rates were significantly lower in patients who were obese versus non-obese, with the influence of BMI being greater on the efficacy of infliximab than on that of adalimumab and etanercept [8]. In contrast, most measures of clinical response to the B cell targeting agent rituximab are unaffected by patient BMI [10]. The impact of body weight or BMI on the response to the interleukin (IL)-6 antagonist tocilizumab is still unclear: whereas baseline body weight was found to influence treatment response in a large trial [11, 12], baseline BMI had no effect in small retrospective studies [13, 14]. Both interventional and real-world studies have found that clinical outcomes with abatacept are not impaired in patients with a BMI > 25 kg/m^2^ [15–19].

Abatacept is a selective T cell co-stimulation modulator that targets the CD80/CD86:CD28 pathway required for full T cell activation. In adults, abatacept is approved for the treatment of moderate-to-severe RA and is available as subcutaneous (SC) and intravenous (IV) formulations [20, 21]. The SC formulation is administered as a fixed weekly 125-mg dose, whereas the IV formulation of abatacept requires a weight-tiered dosing regimen (~ 10 mg/kg every 4 weeks). IV abatacept exhibits a linear pharmacokinetic profile, whereas SC abatacept has a short-term zero-order infusion pattern of absorption [22, 23]. Patient body weight has been shown to influence the clearance of abatacept, emphasizing the importance of a weight-tiered dosing regimen for IV administration [15, 24–27]. Fixed SC dosing achieves trough abatacept serum concentrations comparable to or higher than those observed with IV administration and above the abatacept serum concentration of 10 μg/mL needed for therapeutic effect.

The Abatacept Comparison of subQ versus intravenoUs in Inadequate Responders to mEthotrexate (ACQUIRE) study was a phase 3b, randomized, double-blind, double-dummy study that compared the efficacy and safety of SC and IV abatacept in patients with RA and an inadequate response to ≥3 months of methotrexate treatment [28]. Noninferiority of SC abatacept to IV abatacept was demonstrated; the proportion of patients achieving ≥ 20% improvement in American College of Rheumatology criteria (ACR20) after 6 months was similar across weight categories in the SC and IV groups (with some numeric differences) and also between SC and IV groups within each weight category.

We present findings from a post hoc analysis of the ACQUIRE study. The analysis was designed to complement the findings of the parent study by exploring the impact of patient BMI at baseline on disease activity status after 6 months, measured by stringent remission criteria derived from DAS28 (C-reactive protein; DAS28 [CRP]), Simplified Disease Activity Index (SDAI), and Clinical Disease Activity Index (CDAI). The pharmacokinetics of SC and IV abatacept by patient BMI were also explored.

## Patients and methods

### Study design and patient population

A post hoc analysis of the ACQUIRE (NCT00559585) study was conducted to evaluate the effect of baseline BMI on the pharmacokinetics of and the clinical response to SC or IV abatacept. The study design, ethics approvals, patient population, and inclusion and exclusion criteria have been reported previously [28]. Briefly, patients with active RA and an inadequate response to methotrexate for ≥ 3 months (≥ 15 mg/week) were randomly assigned to receive SC abatacept (125-mg weekly fixed dose) or IV abatacept (~ 10 mg/kg according to body weight range [<60, 60–100, > 100 kg] every 4 weeks). All patients continued methotrexate at the same dose they were receiving at randomization (≥ 15 mg/week). Patients discontinued any other concomitant DMARDs at least 4 weeks prior to randomization; however, they were permitted to continue any concomitant stable low-dose oral corticosteroids (equivalent to ≤ 10 mg/day prednisone) [28].

### Study assessments

All data analyses were performed for both separate and pooled administration routes by baseline BMI subgroup: underweight/normal (< 25 kg/m^2^), overweight (25 to < 30 kg/m^2^), and obese (≥ 30 kg/m^2^).

Patient demographic data and disease characteristics were assessed by BMI subgroup for those with BMI data available at baseline. The clinical response to abatacept by BMI subgroup at month 6 (day 169) was assessed by determining the proportion of patients in remission for the separate and pooled SC and IV groups using each of the following criteria: DAS28 (CRP) <2.6, SDAI score ≤ 3.3, and CDAI score ≤ 2.8. The proportions of patients who achieved an ACR20, ACR50 (≥ 50% improvement), or ACR70 (≥ 70% improvement) response, and mean change from baseline in Patient Global Assessment (PtGA), tender joint count-28 joints (TJC28), and swollen joint count-28 joints (SJC28) at month 6 were also determined.

### Pharmacokinetic analyses

Venous blood samples were collected for the assessment of abatacept steady-state trough plasma concentration (C_minss_) at month 3 (day 85) and month 6 (day 169). It should be noted that blood samples for analysis were obtained prior to abatacept administration. Summary statistics for C_minss_ were presented by route of administration and by BMI subgroup. Abatacept serum concentrations were measured using a validated enzyme-linked immunosorbent assay using colorimetric detection.

### Statistical analyses

Baseline patient demographic data and clinical characteristics were analyzed descriptively according to BMI subgroup (percentage for categorical variables and mean [standard deviation] for continuous variables). The proportions of patients with clinical response over 6 months were presented as pooled data and by route of administration for each BMI subgroup. The rates, mean values, and mean change from baseline for various measures of clinical response were determined with corresponding 95% confidence intervals (CIs) at several time points over 6 months. The median, mean, and first and third quartiles of abatacept C_min_ were presented as a box plot by route of administration and BMI at month 3 and at month 6.

## Results

### Analysis population

Baseline BMIs were available for 1456/1457 patients: 526/1456 (36%) patients were underweight/normal, 497/1456 (34%) were overweight, and 433/1456 (30%) were obese. Baseline demographic data and disease characteristics were similar across BMI subgroups and by route of abatacept administration; hence, the data were also pooled for administration routes (Table 1). Data were obtained for all patients who were either overweight or obese at baseline. For those with an underweight/normal BMI at baseline, data were obtained for 262/265 patients who were administered SC abatacept and for 258/261 patients who were administered IV abatacept.

**Table 1:** Baseline patient characteristics by BMI

	SC abatacept			IV abatacept			Pooled SC/IV abatacept		
	Underweight/ normal BMI *n* = 265	Overweight BMI *n* = 249	Obese BMI *n* = 221	Underweight/ normal BMI *n* = 261	Overweight BMI *n* = 248	Obese BMI *n* = 212	Underweight/ normal BMI *n* = 526	Overweight BMI *n* = 497	Obese BMI *n* = 433
BMI, kg/m^2^	22.0 (2.2)	27.2 (1.4)	35.2 (5.1)	22.0 (2.1)	27.5 (1.5)	35.3 (5.4)	22.0 (2.1)	27.4 (1.4)	35.2 (5.2)
Age, years	46.0 (14.3)	51.4 (12.4)	53.0 (11.4)	49.0 (14.3)	51.2 (12.1)	50.1 (10.7)	47.5 (14.4)	51.3 (12.2)	51.6 (11.2)
Females, %	84.9	78.7	90.0	81.2	79.0	81.1	83.1	78.9	85.7
Caucasians, %	72.1	77.1	75.1	69.7	75.4	79.2	70.9	76.3	77.1
RA duration, years	8.2 (8.0)	7.1 (6.8)	7.6 (9.4)	8.3 (8.3)	8.2 (8.2)	6.2 (6.6)	8.3 (8.2)	7.7 (7.5)	6.9 (8.2)
TJC28	16.4 (6.6)	17.0 (6.4)	17.5 (6.2)	16.3 (6.6)	16.9 (6.4)	17.3 (6.3)	16.3 (6.6)	16.9 (6.4)	17.4 (6.2)
SJC28	14.2 (5.8)	14.0 (5.4)	14.6 (5.5)	13.8 (5.4)	14.2 (5.5)	13.6 (5.1)	14.0 (5.6)	14.1 (5.4)	14.1 (5.3)
hsCRP, mg/dL	3.2 (3.6)	2.5 (2.5)	2.1 (2.3)	3.0 (3.3)	2.5 (2.7)	2.6 (2.8)	3.1 (3.4)	2.5 (2.6)	2.3 (2.5)
HAQ-DI	1.7 (0.7)	1.7 (0.7)	1.8 (0.7)	1.6 (0.7)	1.7 (0.7)	1.7 (0.7)	1.7 (0.7)	1.7 (0.7)	1.8 (0.7)
DAS28 (CRP)	6.3 (0.9)	6.2 (0.9)	6.3 (0.8)	6.2 (0.8)	6.2 (0.9)	6.3 (0.8)	6.2 (0.9)	6.2 (0.9)	6.3 (0.8)
PtGA, 100 mm VAS	67.8 (19.1)	65.8 (20.6)	66.7 (21.6)	64.8 (19.9)	64.0 (19.9)	66.3 (20.2)	66.3 (19.5)	64.9 (20.3)	66.5 (20.9)

Data are mean (standard deviation) unless indicated otherwise

*BMI* body mass index, *CRP* C-reactive protein, *DAS28* Disease Activity Score 28, *HAQ-DI* Health Assessment Questionnaire-Disability Index, *hsCRP* high-sensitivity CRP, *IV* intravenous, *PtGA* patient global assessment, *RA* rheumatoid arthritis, *SC* subcutaneous, *SJC28* swollen joint count-28 joints, *TJC28* tender joint count-28 joints, *VAS* visual analogue scale

### Clinical response to abatacept by BMI subgroup

#### Separate SC and IV group analyses

**Fig. 1:**
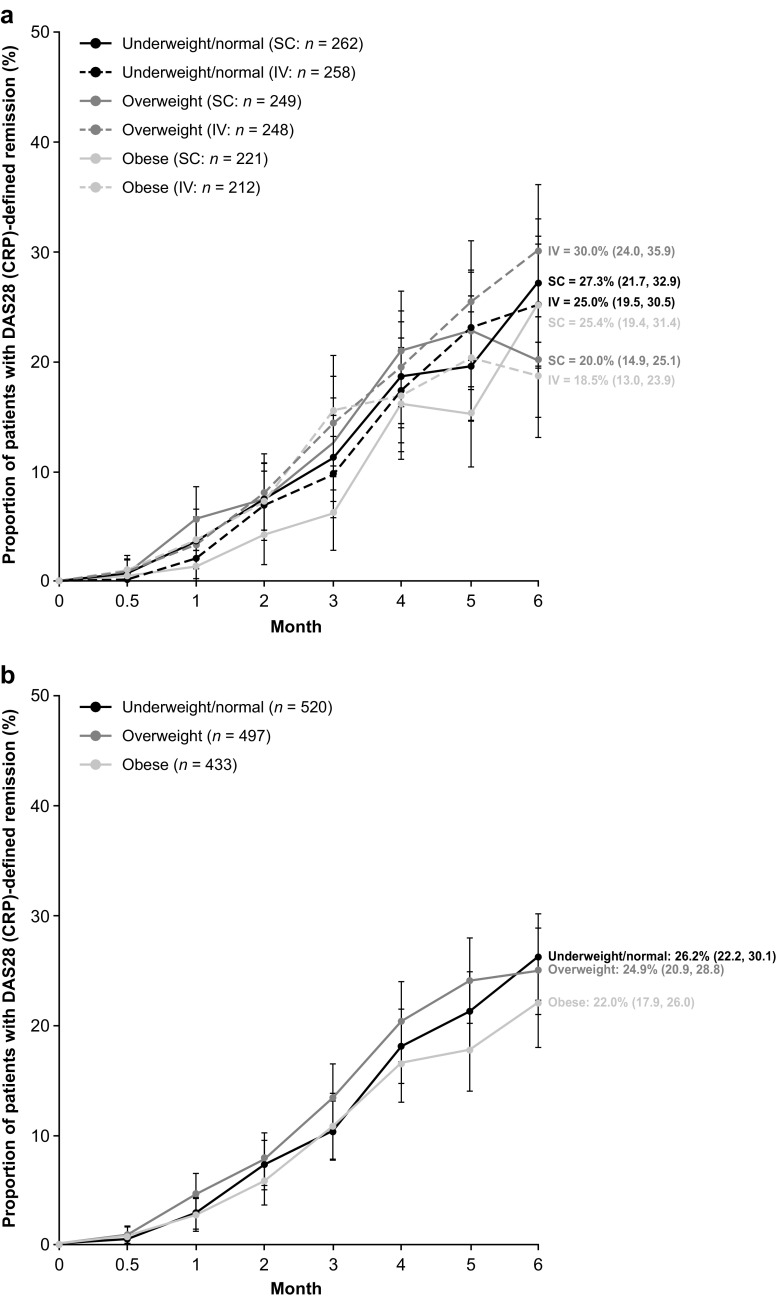
Proportions of patients receiving subcutaneous (SC) or intravenous (IV) abatacept achieving Disease Activity Score 28 (DAS28; C-reactive protein) remission over 6 months by baseline body mass index in **a** the separate analyses and **b** the pooled analysis by route of abatacept administration. As-observed analysis in the intent-to-treat population (> 90% of patients reached the final observation at day 169). *Error bars* represent 95% confidence intervals

**Fig. 2:**
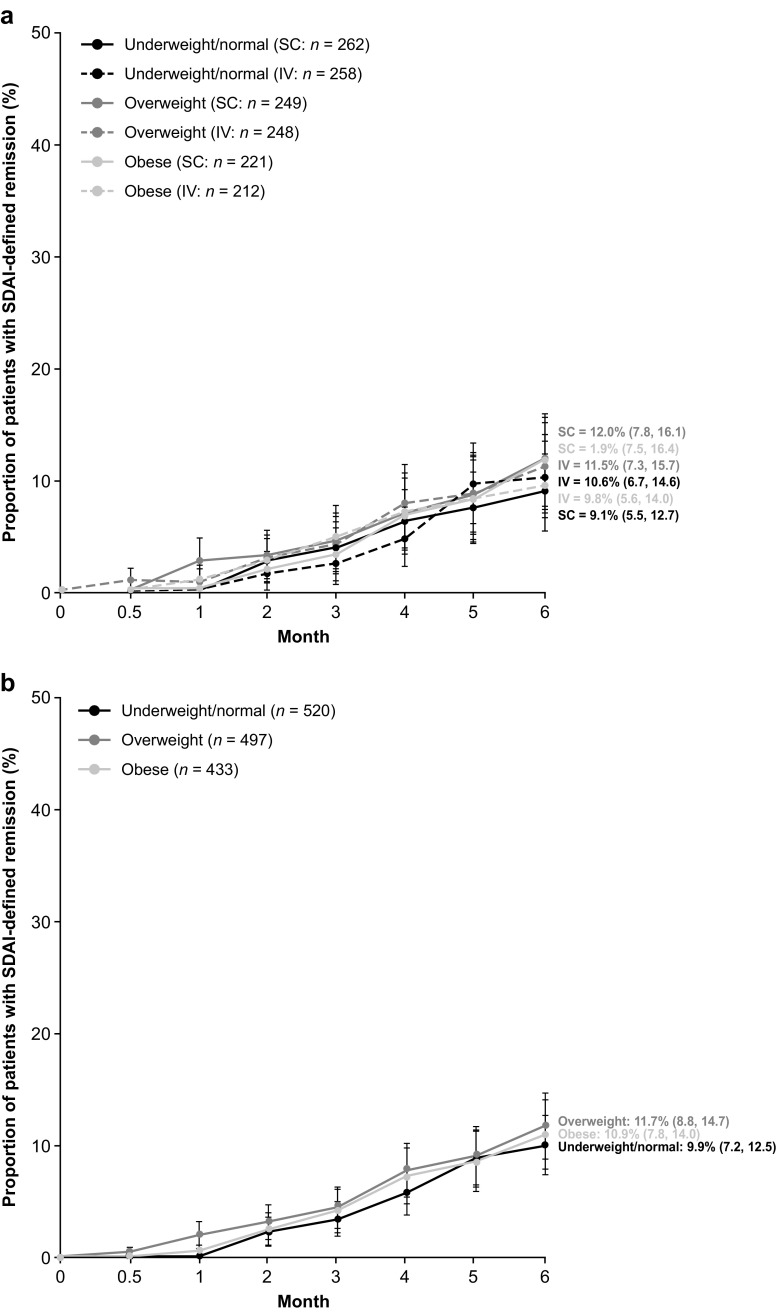
Proportions of patients receiving subcutaneous (SC) or intravenous (IV) abatacept achieving Simplified Disease Activity Index (SDAI) remission over 6 months by baseline body mass index in **a** separate analyses and **b** the pooled analysis by route of abatacept administration. As-observed analysis in the intent-to-treat population (> 90% of patients reached the final observation at day 169). *Error bars* represent 95% confidence intervals

**Fig. 3:**
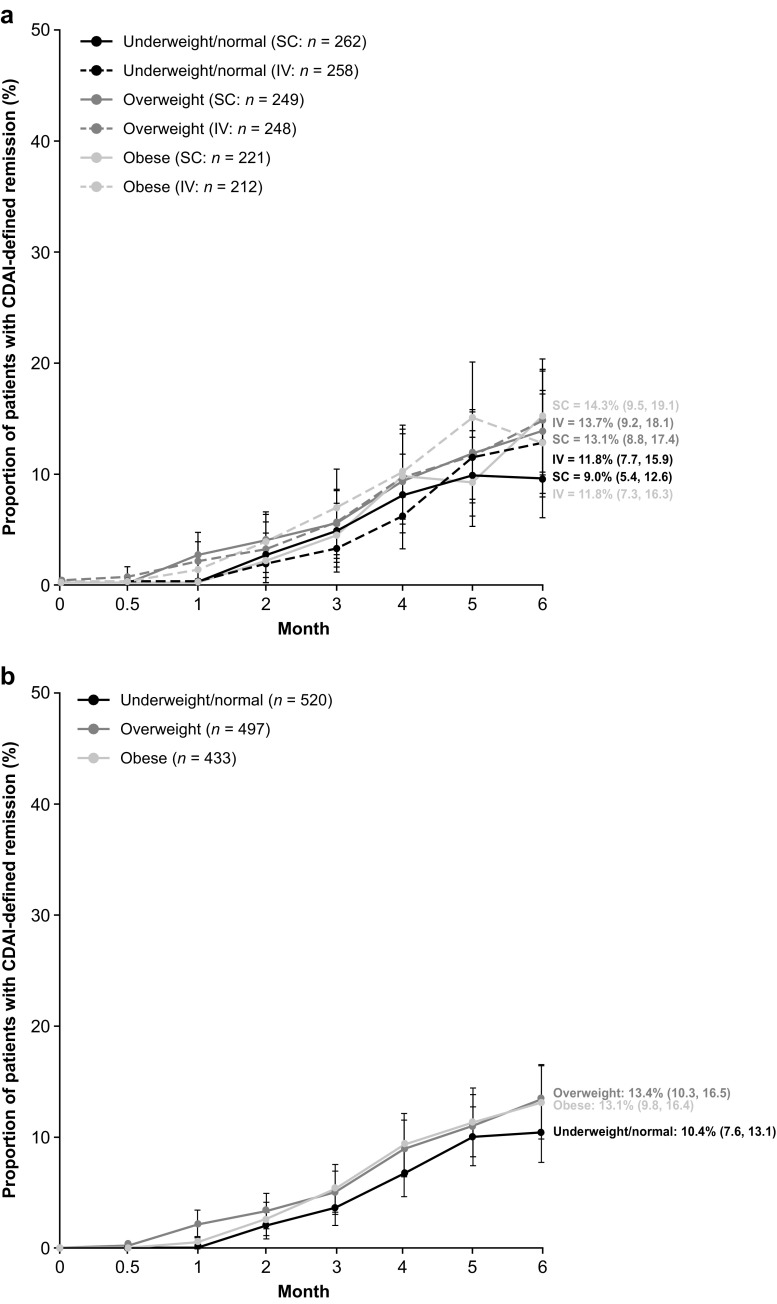
Proportions of patients receiving subcutaneous or intravenous abatacept achieving Clinical Disease Activity Index remission over 6 months by baseline body mass index in **a** the separate analyses and **b** the pooled analysis by route of abatacept administration. As-observed analysis in the intent-to-treat population (> 90% of patients reached the final observation at day 169). *Error bars* represent 95% confidence intervals

There were numerical differences in the proportions (95% CI) of patients achieving DAS28 (CRP) remission at month 6 when stratified by baseline BMI; however, the 95% CIs overlapped across BMI groups (Table 2). For the obese BMI subgroup, DAS28 (CRP) remission (95% CI) was achieved by 25.4% (19.4, 31.4) and 18.5% (13.0, 23.9) of SC and IV abatacept-treated patients, respectively (Table 2; Fig. 1a). SDAI and CDAI remission rates (95% CI) at month 6 were comparable across the BMI subgroups. For the obese BMI subgroup, SDAI remission was achieved by 11.9% (7.5, 16.4) and 9.8% (5.6, 14.0) of SC and IV abatacept-treated patients, respectively, and CDAI remission was achieved by a corresponding 14.3% (9.5, 19.1) and 11.8% (7.3, 16.3) of patients (Table 2; Figs. 2a and 3a). DAS28 (CRP) low disease activity (LDA; DAS28 [CRP] < 3.2) was achieved by 245/268 (91.4%) and 258/272 (94.9%) of SC and IV abatacept-treated patients at month 6, respectively; 375/411 (91.2%) and 353/386 (91.5%) SC- and IV-treated patients, respectively, had DAS28 (CRP) ≥ 3.2. Response rates for ACR20, ACR50, and ACR70 were numerically comparable by BMI subgroup (data reported elsewhere [29]). No significant differences in the mean change from baseline PtGA, TJC28, and SJC28 were observed between BMI subgroups in the separate SC and IV groups (Table 2).

**Table 2:** Clinical response or mean change from baseline at month 6 by BMI

	SC abatacept			IV abatacept			Pooled SC/IV abatacept		
	Underweight/ normal BMI	Overweight BMI	Obese BMI	Underweight/ normal BMI	Overweight BMI	Obese BMI	Underweight/ normal BMI	Overweight BMI	Obese BMI
TJC28	−11.8 (0.4) (−12.6, −11.0)	−11.2 (0.5) (−12.1, −10.3)	−11.9 (0.5) (−12.9, −10.9)	−10.9 (0.4) (−11.7, −10.1)	−11.6 (0.4) (−12.5, −10.8)	−11.5 (0.5) (−12.4, −10.6)	−11.4 (0.3) (−11.9, −10.8)	−11.4 (0.3) (−12.0, −10.8)	−11.7 (0.4) (−12.4, −11.0)
SJC28	−10.3 (0.4) (−11.1, −9.6)	−8.2 (0.4) (−8.9, −7.4)	−10.3 (0.4) (−11.2, −9.5)	−9.7 (0.3) (−10.3, −9.0)	−8.8 (0.4) (−9.6, −8.1)	−9.8 (0.4) (−10.5, −9.0)	−10.0 (0.3) (−10.5, −9.5)	−8.5 (0.3) (−9.0, −8.0)	−10.0 (0.3) (−10.6, −9.5)
hsCRP, mg/dL	−2.0 (0.2) (−2.4, −1.7)	−1.5 (0.2) (−1.8, −1.2)	−0.7 (0.2) (−1.0, −0.4)	−1.9 (0.2) (−2.3, −1.5)	−1.4 (0.2) (−1.8, −1.1)	−1.1 (0.2) (−1.5, −0.7)	−2.0 (0.1) (−2.2, −1.7)	−1.5 (0.1) (−1.7, −1.2)	−0.9 (0.1) (−1.1, −0.6)
DAS28 (CRP) remission, % (95% CI)	27.3 (21.7, 32.9)	20.0 (14.9, 25.1)	25.4 (19.4, 31.4)	25.0 (19.5, 30.5)	30.0 (24.0, 35.9)	18.5 (13.0, 23.9)	26.2 (22.2, 30.1)	24.9 (20.9, 28.8)	22.0 (17.9, 26.0)
SDAI remission, % (95% CI)	9.1 (5.5, 12.7)	12.0 (7.8, 16.1)	11.9 (7.5, 16.4)	10.6 (6.7, 14.6)	11.5 (7.3, 15.7)	9.8 (5.6, 14.0)	9.9 (7.2, 12.5)	11.7 (8.8, 14.7)	10.9 (7.8, 14.0)
CDAI remission, % (95% CI)	9.0 (5.4, 12.6)	13.1 (8.8, 17.4)	14.3 (9.5, 19.1)	11.8 (7.7, 15.9)	13.7 (9.2, 18.1)	11.8 (7.3, 16.3)	10.4 (7.6, 13.1)	13.4 (10.3, 16.5)	13.1 (9.8, 16.4)
PtGA, 100 mm VAS	−36.7 (1.6) (−39.8, −33.6)	−35.8 (1.7) (−39.1, −32.4)	−33.8 (2.0) (−37.7, −29.8)	−34.7 (1.7) (−37.9, −31.4)	−33.8 (1.7) (−37.1, −30.6)	−30.8 (2.0) (−34.8, −26.9)	−35.7 (1.2) (−37.9, −33.4)	−34.8 (1.2) (−37.1, −32.5)	−32.3 (1.4) (−35.1, −29.5)

Data are mean (standard deviation) (95% confidence interval) change from baseline unless indicated otherwise

*CDAI* Clinical Disease Activity Index, *CRP* C-reactive protein, *DAS28* Disease Activity Score 28, *hsCRP* high-sensitivity CRP, *IV* intravenous, *PtGA* patient global assessment, *SC* subcutaneous, *SDAI* Simplified Disease Activity Index, *SJC28* swollen joint count-28 joints, *TJC28* tender joint count-28 joints, *VAS* visual analogue scale

#### Pooled SC and IV analysis

In the pooled SC and IV analysis, the proportions of patients achieving DAS28 (CRP) remission (< 2.6) at month 6 were similar when stratified by baseline BMI (Table 2; Fig. 1b). At month 6, DAS28 (CRP) (95% CI) remission was achieved by 22.0% (17.9, 26.0) of patients in the obese BMI subgroup. SDAI and CDAI remission rates at month 6 were also comparable across BMI subgroups: 13.4% (10.3, 16.5) and 13.1% (9.8, 16.4), respectively, in the obese BMI subgroup (Table 2; Figs. 2b and 3b). No significant differences were observed between BMI subgroups in the mean change from baseline in core components of DAS28, SDAI, and CDAI, namely PtGA, TJC28, and SJC28 (Table 2). The mean change from baseline high-sensitivity CRP was significantly lower in the obese versus underweight/normal and overweight BMI subgroups; however, no impact on remission rates was observed (Table 2).

### Pharmacokinetic analyses

**Fig. 4:**
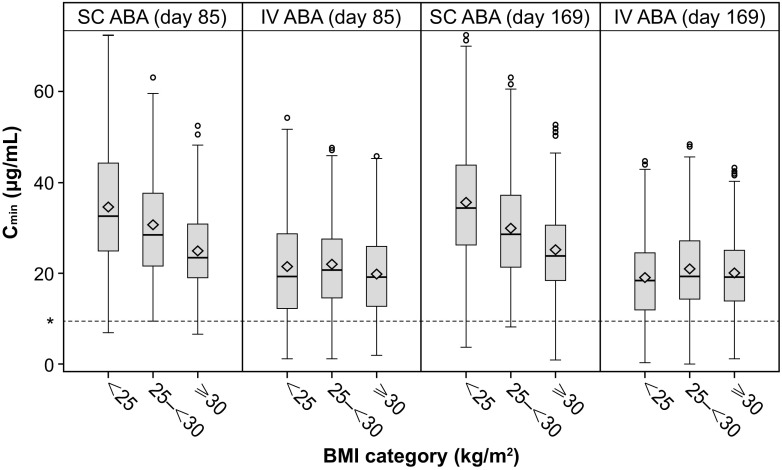
Minimum plasma concentrations (C_min_) of abatacept (ABA) at month 3 (day 85) and month 6 (day 169) by baseline body mass index (BMI) in patients receiving subcutaneous (SC) or intravenous (IV) abatacept *10 μg/mL. *Bottom and top of box* = first and third quartiles; *band inside box* = median; *ends of whiskers* = last observed value within 1.5 times the interquartile range; *diamond* = mean C_min_ > 10 μg/mL is associated with near-maximal efficacy

Steady-state trough concentrations (C_minss_) of abatacept were achieved by month 3 in all patients, consistent with an abatacept half-life of 14 days. Overall, abatacept C_minss_ was higher following the fixed-dose SC administration of 125 mg weekly compared with the body weight-tiered monthly IV administration of 10 mg/kg. A median C_minss_ ≥ 10 μg/mL was achieved in > 90% of patients across all BMI groups for both SC and IV administration. Median C_min_ was numerically higher in the SC than in the IV abatacept group: 33.4, 29.1, and 24.1 μg/mL versus 19.6, 21.3, and 19.7 μg/mL at month 3 and 35.1, 29.0, and 24.1 μg/mL versus 19.1, 20.0, and 19.8 μg/mL at month 6 in the underweight/normal, overweight, and obese BMI subgroups, respectively (Fig. 4). Overall, median abatacept C_min_ was numerically higher with SC versus IV administration for patients with LDA (30.1 vs 22.2 μg/mL at month 3 and 30.9 vs 21.1 μg/mL at month 6, respectively), and for those with DAS28 (CRP) ≥ 3.2 (27.9 vs 19.4 μg/mL at month 3 and 27.8 vs 18.7 μg/mL at month 6, respectively). However, median abatacept C_min_ were similar in patients with and without LDA for IV and SC administration and the ranges were large and overlapping for all subgroups (data not shown).

## Conclusions

This analysis was performed to supplement the findings of the ACQUIRE study, which showed consistent and comparable clinical efficacy of IV and SC abatacept, using ACR response criteria, irrespective of patient body weight. In this post hoc analysis, the more stringent outcomes of remission with stratification by BMI showed consistent clinical efficacy of SC and IV abatacept irrespective of baseline patient BMI. Therapeutic abatacept plasma trough concentrations were achieved in > 90% of patients with both routes of administration and the trend towards a higher C_min_ with lower BMI observed with SC administration was not associated with differences in remission rates.

The results reported here are consistent with previous findings from both real-world and interventional studies with abatacept [15–18, 30]. In the real-world ACTION study and a pan-European analysis of pooled RA registry data, good/moderate European League Against Rheumatism (EULAR) responses were achieved across all BMI subgroups [16, 30]. Similarly, a post hoc analysis of data from the APPRAISE study showed that baseline BMI did not influence the effect of abatacept on synovitis detected by the objective measure of power Doppler ultrasonography [17]. These findings contrast with the evidence for TNFis, which indicates reduced efficacy in patients with higher BMI [9, 31]. This difference between abatacept and TNFis could be attributable to their different modes of action. Visceral fat may induce resistance to or neutralization of TNFis [9], and increased levels of pro-inflammatory cytokines may alter the distribution and pharmacokinetics of TNFis, although current evidence is inconclusive [3]. The effect of patient body weight or BMI on clinical response to tocilizumab is unclear [11, 14, 32].

In vitro experiments have shown that abatacept concentrations of 10 μg/mL are associated with maximal T cell inactivation [33]. Consistent with this, a C_minss_ of > 10 μg/mL is associated with near-maximal efficacy of abatacept [15], including in terms of reduction in DAS28 (CRP) in exposure–response analyses (data on file). In this post hoc analysis, a C_minss_ of > 10 μg/mL was attained in most patients, irrespective of BMI and the pharmacokinetic differences observed between IV and SC dosing. As expected, a more homogeneous distribution of median abatacept C_minss_ values across BMI groups was achieved with the weight-tiered IV dosing than with the SC route of administration. The decrease in median observed C_min_ values with increased patient BMI with the fixed-dose SC administration is likely to be due to a more rapid clearance of abatacept at increased body weight. However, despite these pharmacokinetic differences between IV and SC dosing, we found therapeutic exposures were achieved across all BMI groups with both routes of administration, with no significant relationship between the median C_min_ and clinical remission rates with SC abatacept [26]. Exposure–ACR20 response modeling suggested IV abatacept doses above 10 mg/kg and a C_minss_ above > 10 μg/mL are unlikely to bring additional clinical benefit [34]. In this analysis, the range of C_min_ was broad and similar for patients with and without DAS28 (CRP) LDA with either SC or IV administration. The higher median abatacept C_min_ with SC versus IV dosing was not associated with higher rates of DAS28 (CRP) LDA. Small patient numbers precluded further subgroup analyses to determine the impact of BMI on the association between C_min_ and clinical efficacy. In recent studies of other bDMARDs, optimal trough levels of either adalimumab (fixed dose) or infliximab (weight tiered) were similarly achieved across BMI subgroups, with a trend towards lower adalimumab plasma trough levels in patients with a BMI > 30 kg/m^2^; the effect on clinical outcomes, however, was not investigated [35, 36].

The potential for individual patient characteristics to modify clinical effect, patient preference for route of administration, and treatment pharmacoeconomics are all considerations in the development of a personalized treatment plan [37]. The stratification of patients by BMI and the use of stringent remission criteria in this post hoc analysis provides evidence that for obese patients with RA, in whom response rates with bDMARDs may be suboptimal [38], the SC and IV formulations of abatacept could be considered equally, and could also help to reduce the additional risk of RA-associated cardiovascular morbidity in the long term [39–41].

In addition to the inherent limitations of a post hoc analysis, other limitations should be considered. The relationship between body fat and bDMARDs such as abatacept is not fully understood and requires further investigation. This study was an analysis of data from the 6-month double-blind period of the primary ACQUIRE study only; however, response rates to abatacept were maintained regardless of baseline body weight in the long-term extension study of ACQUIRE, in which all patients were switched from IV to SC abatacept [42].

In summary, SC and IV abatacept demonstrated a comparable clinical efficacy, using stringent remission criteria, which was independent of baseline BMI. Most patients achieved therapeutic plasma concentrations of abatacept, irrespective of route of administration. Abatacept could be considered an appropriate treatment option for patients with RA regardless of BMI status.
